# Loss of CENP-I Impairs Homologous Recombination and Sensitizes Cells to PARP1 Inhibition

**DOI:** 10.3390/cancers13133202

**Published:** 2021-06-26

**Authors:** Tuyen T. Dang, Julio C. Morales

**Affiliations:** Stephenson Cancer Center, Department of Neurosurgery, University of Oklahoma Health Science Center, Oklahoma City, OK 73104, USA; Tuyen-Dang@ouhsc.edu

**Keywords:** CENP-I, homologous recombination, PARP1, GBM

## Abstract

**Simple Summary:**

Centromere Protein I (CENP-I) is one of a family of cellular molecules that is essential for the separation of chromosomes during mitosis and mitotic progression. Interestingly, other CENP family proteins, such as CENP-A, have been shown to be involved in double-strand break repair. The goal of the current study was to determine the role of CENP-I in DNA repair and the DNA damage response. We found that loss of CENP-I results in increased double-strand break formation and chromosomal aberration. Consistent with these results we found that loss of CENP-I impairs homologous recombination and sensitizes cells to PARP1 inhibition. We also found that CENP-I expression is elevated in patients suffering from glioblastomas, suggesting that CENP-I may play a role in the progression for this disease.

**Abstract:**

Centromere Protein I (CENP-I) is a member of the CENP-H/I/K complex. CENP-H/I/K is a major component of the inner kinetochore and aids in ensuring proper chromosomal segregation during mitosis. In addition to this chromosomal segregation function, CENP-I also plays a role in DNA double-strand break (DSB) repair. Loss of CENP-I leads to increased endogenous 53BP1 foci and R-loop formation, while reducing cellular survival after ionizing radiation and Niraparib, a PARP1 small molecule inhibitor, exposures. Cells lacking CENP-I display delayed 53BP1 foci regression, an indication that DSB repair is impaired. Additionally, loss of CENP-I impairs the homologous recombination DSB repair pathway, while having no effect on the non-homologous end-joining pathway. Interestingly, we find that RNaseH1 expression restores HR capacity in CENP-I deficient cells. Importantly, CENP-I expression is elevated in glioma tissue as compared to normal brain tissue. This elevated expression also correlates with poor overall patient survival. These data highlight the multi-functional role CENP-I plays in maintaining genetic, as well as chromosomal, stability and tumor survival.

## 1. Introduction

Chromosomal segregation is a fundamental process that occurs during every cell division. Accurate and efficient chromosomal segregation is essential for maintaining normal cellular homeostasis, as mistakes in this process often lead to diseased states such as cancer [1]. Mitotic cells are extremely sensitive to DNA damage, especially DNA double-strand breaks (DSBs) [2]. DSBs can be introduced into mitotic cells through a variety of different methods. DSBs introduced into the genome through replication stress can oftentimes be carried over into mitosis [3]. In fact, it is estimated that 10–20 DSBs can bypass the G2/M checkpoint [4]. In addition, while the chromosomal condensation that occurs during mitosis in preparation for segregation is typically protective, if DNA damage is already present, this process can hinder DSB repair [5].

Homologous recombination (HR) or non-homologous end-joining (NHEJ) are the two pathways that primarily repair DSBs [6]. These repair pathways are accompanied by an extensive signaling cascade that aids in the repair process [6]. Nevertheless, mitotic cells undergo an annotated DNA damage response (DDR) [7,8]. In fact, full DDR activation in mitosis can lead to whole chromosomal instability by stabilizing kinetochore/microtubule attachments [9].

The kinetochore is composed of multiprotein complexes that ensures proper chromosomal segregation. One of these protein complexes is the Centromere Protein-H/I/K complex (CENP-H/I/K) [10]. This protein complex directly coordinates kinetochore assembly [10]. Interestingly, several CENP family members have DDR connections. CENP-F aids in ATR (Ataxia Telangiectasis and Rad3-related protein), a key DDR signaling molecule, localization to the centromere [11]. CENP-A is recruited to DSB sites and protects against ionizing radiation exposure [12]. CENP-S and CENP-X have been shown to be involved in both HR and NHEJ repair [13]. However, nothing is known about whether CENP-H, I or K plays a role in DSB repair. In addition, CENP-A has also been shown to regulate RNA:DNA hybrid (R-loop) formation [14].

The aim of this study was to determine the role of CNEP-I in the DNA damage response and double-strand break repair. Our data suggest a role for CENP-I in repairing DNA damage and protecting tumor cells against genotoxic stress.

## 2. Results

### 2.1. CENP-I Expression Is Elevated Gliomas as Compared to Normal Brain

**Figure 1:**
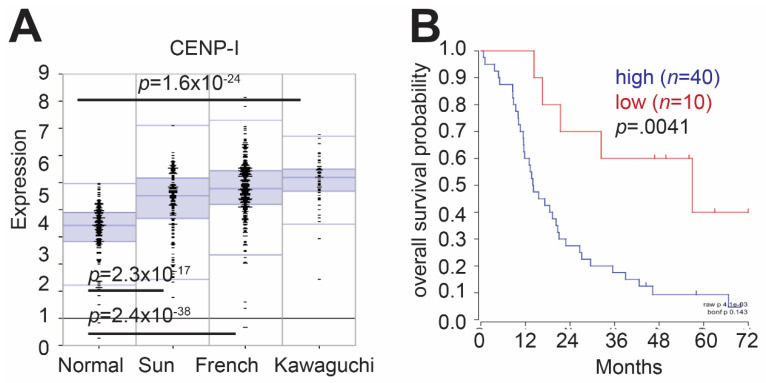
Expression of CENP-I is elevated in glioma patient samples and correlates with poor overall survival. (**A**) CENP-I mRNA levels in normal and tumor datasets. The Sun dataset compared to normal brain has a *p* value = 2.3 × 10^−17^, the French dataset compared to normal brain has a *p* value = 2.4 × 10^−38^ and the Kawaguchi dataset compared to normal brain has a *p* value = 1.6 × 10^−24^. (**B**) Overall patient survival comparing high CENP-I expression (blue line) and low CENP-I expression (red line). Data were accessed through the R2 Genomic and visualization platform. Default settings provided by the platform were used.

CENP-I is a subunit of the CENP-H/I/K complex that makes up the inner kinetochore and safeguards proper kinetochore construction [10]. Using the R2: Genomic Analysis and Visualization Platform (http://r2.amc.nl accessed on 1 June 2020), we examined microarray data from glioma patients provided by the Kawaguchi (GEOID: gse43378) [15], Sun (GEOID: gse4290) [16] and French (GEOID: gse16011) [17] datasets. We found that CENP-I expression is elevated in glioma tumor samples, as compared to normal brain tissue (Figure 1A). Using the Kawaguchi dataset, we found that elevated CENP-I expression correlated with poor overall patient survival as compared to patients with lower CENP-I expression (Figure 1B). The R2 platform Kaplan Scan feature was used to develop a Kaplan–Meier curve. This feature uses statistical testing instead of an average or median expression cutoff to separate I into high or low CENP-I expressing groups.

### 2.2. Loss of CENP-I Sensitizes Cells to Genotoxic Stress

**Figure 2:**
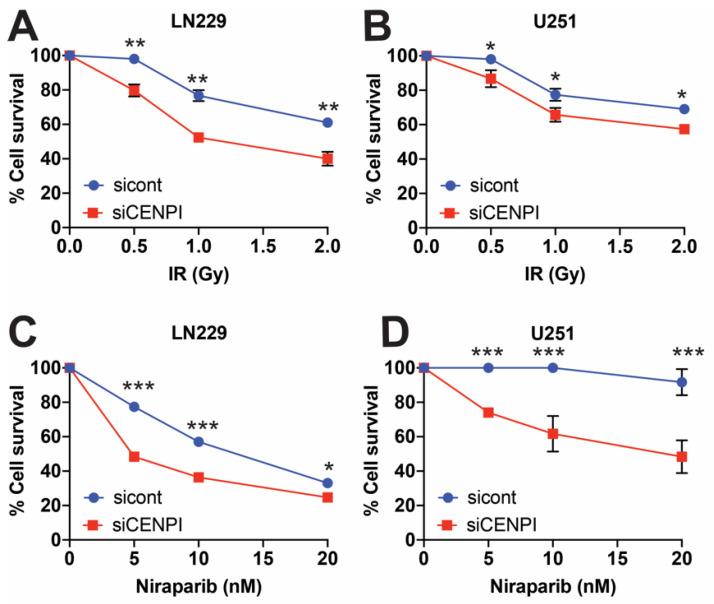
Loss of CENP-I sensitizes cells to genotoxic stress. (**A**,**B**) LN229 and U251 cells with and without CENP-I were exposed to indicated doses of ionizing radiation (IR). (**C**,**D**) LN229 and U251 cells were treated with indicated doses of Niraparib. Statistical analysis was performed using Student’s *t*-test. * = *p* ≤ 0.05, ** *= p* ≤ 0.01, *** = *p* ≤ 0.001.

To gain a better understanding on how loss of CENP-I affects cells, we ablated CENP-I expression in U251 and LN229 cells using three non-overlapping CENP-I or control siRNAs (Supplemental Figure S1A–C). We then treated CENP-I and control siRNA-exposed cells with ionizing radiation (IR) or Niraparib, a PARP1 small molecule inhibitor [18]. Loss of CENP-I sensitized LN229 and U251 cells to IR (Figure 2A,B) and Niraparib (Figure 2C,D) exposure. Interestingly, loss of CENP-I sensitized cells to IR similar to loss of PTEN [19], while the sensitization to Niraparib was much more significant. These results suggest that CENP-I is important for HR because increased sensitivity to IR can come about due to defects in the NHEJ or HR pathways, while sensitivity to PARP1 inhibition is due to a defect in HR repair, and loss of NHEJ results in resistance [20]. Importantly, we found a minimal difference in relative cell number between cells transfected with CENP-I siRNAs as compared to control (Supplemental Figure S2).

Increased sensitivity to IR exposure is commonly associated with a defect in DSB repair [21,22,23,24]. Decreased cellular survival in response to PARP1 inhibition is commonly associated with defects in HR repair [25]. Our data suggest that CENP-I is involved in the DNA damage response (DDR), and DSB repair is through the HR repair pathway.

### 2.3. Loss of CENP-I Results in Increased 53BP1 Foci and Delayed Repair Kinetics

**Figure 3:**
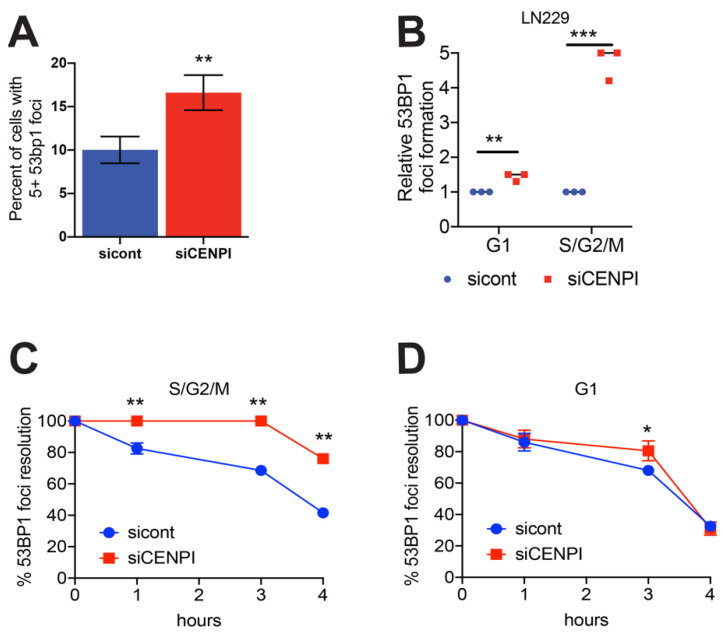
Loss of CENP-I results in increased 53BP1 foci. (**A**) 53BP1 foci were examined in LN229 cells with and without CENP-I. (**B**) 53BP1 foci were measured in G1 and S/G2/M LN229 cells. (**C**,**D**) 53BP1 foci were measured in G1 and S/G2/M at 1, 3 and 4 h after exposure to ionizing radiation (1 Gy). Statistical analysis was performed using Student’s *t*-test. * = *p* ≤ 0.05, ** *= p* ≤ 0.01, *** *= p* ≤ 0.001.

As loss of CENP-I resulted in decreased cellular survival after genotoxic stress, we examined whether CENP-I is involved in DSB repair. We examined 1000 cells transfected with control or CENP-I siRNAs for DSB formation using 53BP1 foci formation as a marker [26]. Indeed, we found that loss of CENP-I resulted in increased 53BP1 foci formation. Loss of CENP-I resulted in ~15% of an asynchronously growing cell population displaying 5+ 53BP1 foci as compared to ~9% of control cells (Figure 3A, Supplemental Figure S3). Next, we examined whether there was a cell-cycle dependence in the increase in 53BP1 foci formation after CENP-I loss. To do this, we labeled LN229, U251 and G55 glioblastoma cells using the FUCCI (Fluorescent Ubiquitination-base Cell Cycle (Takara Bio, Mountain View, CA, USA) system (Supplemental Figure S4) [27]. This system employs an RFP fused Cdt1 and an AmCyan fused Geminin to indicate whether cells are in G1 or S/G2/M phases of the cell-cycle, respectively. Using this system, we found a ~1.5-fold increase in 53BP1 foci formation in G1, but a ~4-fold increase in 53BP1 foci formation in S/G2/M cells (Figure 3B). Similar results were also found in G55 human GBM cell lines (Supplemental Figure S5). In addition, we examined the resolution of 53BP1 foci in G1 and S/G2/M cells and found that loss of CENP-I resulted in slowed DNA repair kinetics in S/G2/M cells (Figure 3C), but not G1 cells (Figure 3D).

### 2.4. Loss of CENP-I Leads to Increased Chromosomal Aberrations

**Figure 4:**
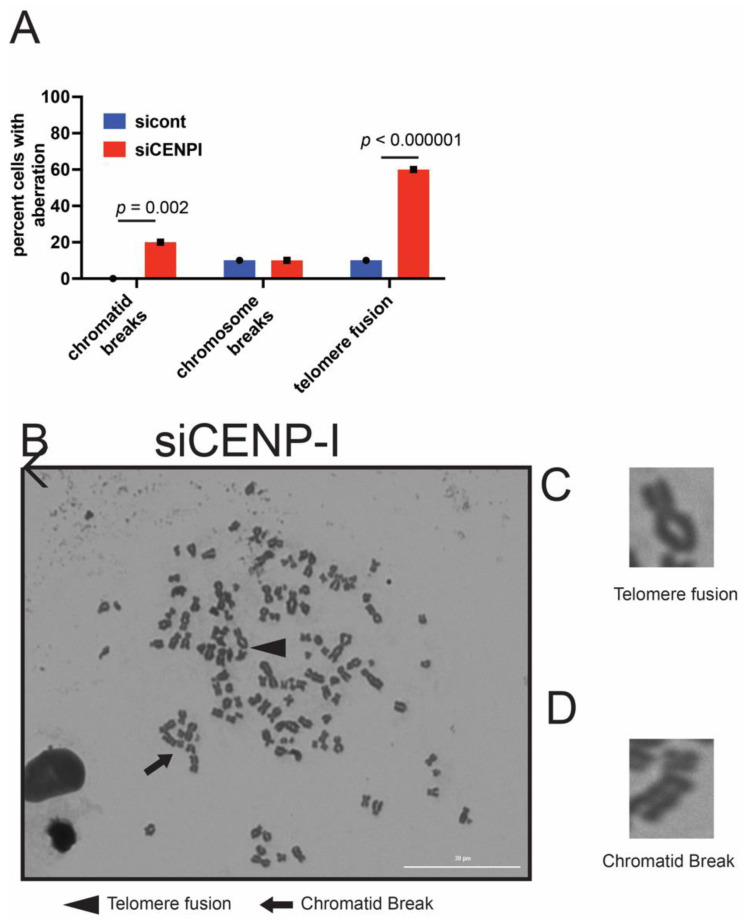
Loss of CENP-I results in increased chromatid breaks and telomere fusions. (**A**–**D**) Chromatid breaks and telomere fusions were visualized and quantitated using CENP-I-deficient and proficient cells using metaphase spreads. Statistical analysis was performed using Student’s *t*-test.

We performed metaphase spreads to examine the extent of genomic instability in CENP-I-deficient cells as compared to control cells. We noted that CENP-I-deficient cells harbored increased amounts of chromatid breaks and telomere fusions as compared to control cells (Figure 4A–D). We found no difference in the amount of chromosome breaks (Figure 4A). As chromatid breaks and telomere defects are commonly associated with loss of HR repair, this result is consistent with our previous observations that loss of CENP-I affects DSB in S and G2 phases of the cell-cycle where HR DSB repair is utilized.

### 2.5. Loss of CENP-I Inhibits HR, But Is Dispensable for NHEJ DSB Repair

**Figure 5:**
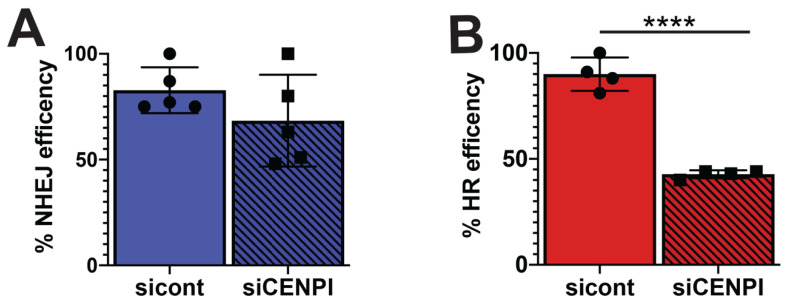
Loss of CENP-I impairs HR repair efficiency. (**A**) Non-homologous end-joining (NHEJ) or (**B**) homologous recombination (HR) repair efficiency was measured in the EJ5 and DR U2OS reporter cell lines exposed to control (cont) or CENP-I siRNA. For each experiment, NHEJ and HR repair efficiency was determined by counting 10,000 cells per condition and normalizing to sicont-treated cells. Statistical analysis was performed using Student’s *t*-test. **** = *p* ≤ 0.0001.

We utilized EJ5 and DR U2OS GFP reporter cells to determine how loss of CENP-I affects HR and NHEJ repair. The EJ5 reporter cell line was used to measure how loss of CENP-I affects total NHEJ repair, while the DR cell line measures HR repair [28]. Expression of GFP indicates a successful repair event. EJ5 and DR cells were transfected with control or CENP-I siRNAs. Interestingly, while we did not observe a significant loss in NHEJ repair efficiency (Figure 5A), we found a ~50% decrease in HR repair (Figure 5B). These data are consistent with our observation that loss of CENP-I results in increased cell death in response to PARP1 inhibition (Figure 2C,D) and abrogated DSB repair kinetics specifically in S/G2/M cells, but not G1 cells (Figure 3C,D).

### 2.6. Loss of CENP-I Results in Increased R-Loop Formation

**Figure 6:**
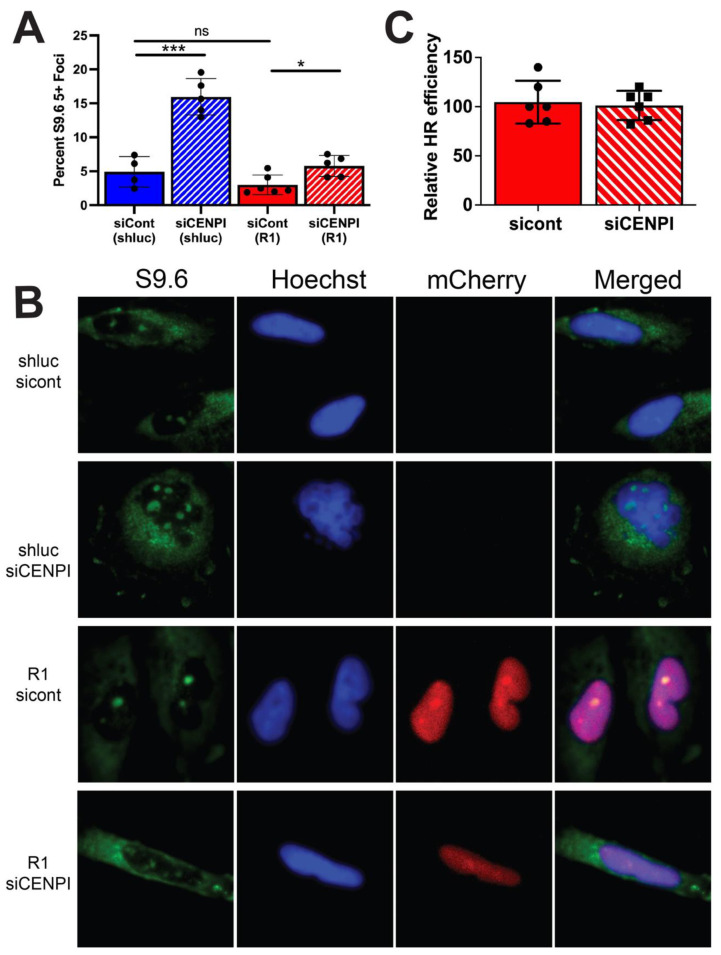
Loss of CENP-I results in RNA:DNA hybrid formation. (**A**,**B**, Supplemental Figure S6) Formation of S9.6 nuclear foci was examined in LN229 control (luc) and LN229 RNaseH1 expressing (R1) cells transfected with control (cont) or CENP-I siRNAs. For each experiment, at least 500 cells were examined. (**C**) Homologous recombination (HR) repair efficiency was measured in the DR U2OS reporter cell lines expressing exogenous RNaseH1 exposed to control (cont) or CENP-I siRNA. For each experiment, HR repair efficiency was determined by counting 10,000 cells per condition and normalizing to sicont-treated cells. Statistical analysis was performed using a Student’s *t*-test. * = *p*< 0.05, *** = *p* ≤ 0.0001.

Recently, several publications have shown the connection between extended RNA:DNA hybrids (R loops) and the DNA damage response, as increased R-loop formation has been shown to result in increased 53BP1 foci and adversely affect DSB repair [29,30,31]. In addition, it has been observed that loss of CENP-A, an interacting partner of CENP-I, results in increased R-loop formation [14]. Thus, we examined whether loss of CENP-I had an effect on R-loop formation. We transfected LN229 GBM cell lines with control or CENP-I siRNA. We examined these cells by immunofluorescence and found that loss of CENP-I resulted in an increase in cells with 5+ foci formation of nuclear S9.6 staining as compared to control (Figure 6A,B, Supplemental Figure S6). Five or greater S9.6 foci was used as our cut-off for R-loop positive cells, as we have shown that loss of XRN2, a factor known to regulate R-loop formation, results in an average of five S9.6 foci per nuclease [31]. To determine that the increase in S9.6 foci was due to an increase in RNA:DNA hybrids, we performed the same experiments in LN229 cells expressing human RNaseH1 [30] and found a reduction in cells with 5+ foci formation of S9.6 nuclear staining (Figure 6A,B). In addition, consistent with recently published data, we found no difference in the level of S9.6 staining in RNaseH1-expressing and non-expressing cells exposed to control siRNA [32]. The decrease in S9.6 foci formation in RNaseH1-expressing cells exposed to CENP-I siRNA as compared to non-RNaseH1-expressing cells is suggestive of formation of genomic R loops.

We previously found that RNA:DNA hybrids can impair DNA repair [30]; thus, we examined whether RNA:DNA hybrids contributed to the HR impairment in CENP-I deficient cells. We transfected previously described RNaseH1-expressing U2OS-DR cells [30] with control and CENP-I siRNAs and found no difference in HR repair efficiency (Figure 6C). Thus, the expression of RNaseH1 restored HR repair capacity to CENP-I-deficient cells.

## 3. Discussion

The timely and effective repair of DSBs is critical to cellular survival and avoiding possible diseased states such as cancer. Thus, to gain a better understanding of how the genome is preserved, it is important to uncover the cellular factors important in DSB repair. Our data provide evidence for CENP-I’s role in DSB repair and protection against genotoxic stress.

### 3.1. Involvement of CENP-I and Homologous Recombination Repair

Similar to loss of 53BP1 and γH2AX, loss of CENP-I decreases cellular survival after genotoxic stress. Cells lacking CENP-I demonstrated increased sensitivity to PARP1 inhibition, which is commonly correlated with a defective HR repair pathway [25]. Loss of CENP-I also sensitizes cells to IR, commonly associated with defects in the HR or NHEJ repair pathways [21,29,31,33,34]. These data suggest a more functional role in DSB repair for CENP-I.

The delay in DSB foci repair and increased sensitivity to DNA damaging agents is often associated with a functional loss in the NHEJ or HR repair pathways [29,31,34]. Interestingly, loss of CENP-I primarily impairs HR repair while being dispensable for NHEJ repair. These data are supported by the fact that we observe defects in 53BP1 foci regression in S/G2/M, where HR repair is the primarily utilized pathway for DSB repair and NHEJ is primarily employed during G1 of the cell-cycle [35].

Similar to CENP-A, we found that loss of CENP-I also results in RNA:DNA hybrid formation [14]. This is significant as RNA:DNA hybrids have been shown to impair RPA recruitment to DSB sites [36]. We also find that one role of CENP-I in HR repair is RNA:DNA hybrid resolution, as expression of RNaseH1 restores HR repair efficiency. This is in contrast to XRN2, where RNaseH1 expression does not restore HR repair efficiency and is implicated in transcription regulation rather than solely RNA:DNA hybrid resolution [30]. Interestingly, as CENP-I is required for CENP-A assembly [37], our data suggest that there is a similar relationship between the two in regards to DNA repair and RNA:DNA hybrid regulation functions.

### 3.2. Therapeutic Potential of CENP-I Targeting

The five-year survival rate for patients suffering from GBMs is less than one year [38]. This survival rate has not changed in 50 years [39]. Therapeutic resistance is one of the major factors contributing to the low GBM patient survival rate [40,41]. A key factor in GBM therapeutic resistance, whether it is acquired or intrinsic, is upregulation of the DNA repair factors [42,43]. Thus, uncovering factors involved in DSB repair in GBMs will allow us to gain an understanding of therapeutic resistance in this disease. We found that CENP-I expression is elevated in glioma patients, and this elevation is associated with poor overall survival. The question now is: why is CENP-I up-regulated? As CENP-I expression is required during mitosis, the elevated expression of CNEP-I in GBMs may be due to the heightened proliferation in the tumor. A second possibility for elevation of CENP-I is to protect the tumor from RNA:DNA hybrid formation. RNA:DNA hybrids are known to act as replication blocks and are not resolved in a timely manner, resulting in genomic instability [44]. Formation of RNA:DNA hybrids can also affect DSB repair kinetics, as unresolved loops impair regression of 53BP1 foci after IR [29]. Our data demonstrate that loss of CENP-I results in impaired DSB repair after IR exposure. As radiation is one of the primary therapeutic agents utilized in GBM therapies, molecular targeting of CENP-I may be useful in enhancing tumor cell killing after IR treatment.

## 4. Materials and Methods

Gene expression and overall survival probability: Gene expression analysis was performed using the R2 genomics analysis and visualization platform (http://r2.amc.nl, accessed on 1 June 2020) (Amsterdam, The Netherlands). Default settings were used to compare expression of CENP-I in a set of normal brain samples (*n* = 172, Berchtold set) to adult glioma samples (*n* = 153, Sun set; *n* = 40, Kawaguchi set; *n* = 284, French set). Overall survival probability for CENP-I was determined using default settings applied to the Kawaguchi data set. Clinical information and how glioma samples were collected for the Sun, French and Kawaguchi datasets have been previously described [15,16,17].

Cell Culture: Cells were maintained at a humidified 37 °C supplemented with 5% CO_2_. G55 and U2OS cells were grown in 10% fetal bovine serum (FBS), 1X Penicillin/Streptomycin (P/S) in DMEM. LN229 cells were grown in 5% FBS, 1X P/S in DMEM. U251 cells were grown in 10% FBS, 1X P/S, 4 mM L-glutamate, 1 mM sodium pyruvate in MEM. All media and media supplements were from Sigma Aldrich (St. Louis, MO, USA).

Transfection: Cells were reverse-transfected with Mission siRNA transfection reagent (Sigma Aldrich, St. Louis, MO, USA, cat no S1452) at 0.2 μL reagent/200 μL total volume. SiRNAs were transfected at 0.5 nM (survival studies), 25 nM (metaphase studies), or 50 nM (Western blot and I-SecI repair studies). Transfections were allowed to go for a minimum of 72 h before end of study.

SiRNAs were purchased from Sigma Aldrich:

siCENPI-01: CAAACCAUUUCGUGUGAGA
siCENPI-02: CCAUGAACUCUGUGUCUAA
siCENPI-03: GAAUUAAAUCACUAUUGA

Western Blot/Immunofluorescence: Western blots were conducted as previously described. Briefly, cells were lysed in RIPA buffer, ran on 10% SDS-page gels, and then transferred on fluorescence compatible PVDF membrane. Samples were probed with CENPI antibody (Thermo Fisher, Waltham, MA, USA, cat no PA566375) and beta-actin (Thermo Fisher, city, state abbreviation, USA, cat no MA515739) overnight. Immunofluorescence was conducted as previously described. Briefly, samples were fixed with 2% formaldehyde, permeabilized with 0.1% TritonX-100 and incubated at RT with 53bp1 antibody (Thermo Fisher, Waltham, MA, USA, cat no PA116566) overnight.

Metaphase: After 72 h transfection, cells were treated with 0.1 μg/mL of Colcemid (Thermo Fisher, USA, cat no15212012) diluted in growth media overnight before processing. Cells were harvested from plates by shake-off method, pelleted with cold PBS and then swelled with 0.075 M KCl for 8 min at RT. Fixing solution of 75% methanol: 25% acetic acid was added to the solution; samples were then spun and resuspended in PBS. Samples were pelleted and resuspended in fixing solution before dropping onto slides. Slides were allowed to dry before staining and imaging.

I-SecI-based DNA repair assays: Experiments were carried out as described with slight modifications (Gunn, Stark. Methods in Molecular Biology, 2012) [28]. Briefly, 24 h after reverse transfection with siRNAs, I-SecI plasmid was transfected into the cells, with no additional amount of siRNA needed. Media were replaced 24 h after plasmid transfection. At 72 h post-plasmid transfection cells were fixed with 2% formaldehyde in PBS and counterstained with Hoechst. Cells were imaged for Hoechst and GFP signals. Percent efficiency was determined by percent cells positive for GFP and Hoechst versus Hoechst alone.

Survival studies: A total of 400–500 cells per well in a 96-well plate were transfected for 24 h before IR treatment using X-Rad 320 irradiator (Precision, North Brandford, CT, USA) or listed drug treatment. Plates were harvested 7–8 days post-transfection and stained with Hoechst for fluorescence intensity readout.

Imaging/Fluorescent intensity: Imaging and plate reading was done on a Cytation 5 instrument (Biotek, Winooski, VT, USA). The following objectives were used: 10X phase Plan Fluorite WD 10 NA 0.3 and 20X phase Plan Fluorite WD 6.7 NA 0.45. Spot counting is a proprietary application within Gen5.

Fucci Cells: Reporter plasmids, pRetroX-G1-Red and pRetroX-SG2M-Cyan (Takara, Mountainview, CA, USA), were forward transfected into 293GP cells with the appropriate packaging vectors to produce retroviruses. Recipient cells were then infected with the viruses and selected based on manufacturer’s instructions.

## 5. Conclusions

In this study, we identified a role for CENP-I in DNA damage response and HR repair. Loss of CENP-I impairs HR DSB repair. On a cellular level, this loss of HR manifests itself as sensitivity to ionizing radiation and Niraparib, a PARP1 inhibitor. Interestingly, expression of CENP-I is elevated in brain-derived tumors (Figure 1). These results suggest that molecular targeting of CENP-I would be beneficial for brain-derived or other tumor types that utilize ionizing radiation or PARP1 inhibitors, such as breast tumors, as common therapies.

## Data Availability

Public data sources are listed in methods.

## Supplementary Materials

The following are available online at https://www.mdpi.com/article/10.3390/cancers13133202/s1, Figure S1: Confirmation of CENP-I expression loss, Figure S2: CENP-I loss does not affect cell growth, Figure S3: Loss of CENP-I induces 53BP1 foci, Figure S4: Generation of cell-cycle reporter GBM cell lines. Figure S5: Loss of CENP-I results induces 53BP1 foci in S/G2/M, Figure S6: Loss of CENP-I results in increased RNA:DNA hybrids.
