# Supplementary material for: Loss of CENP-I Impairs Homologous Recombination and Sensitizes Cells to PARP1 Inhibition
Source: Cancers (Basel). 2021 Jun 26;13(13):3202. doi: 10.3390/cancers13133202 (PMC8267748; doi:10.3390/cancers13133202)
Supplement: Supplementary file 1 [file cancers-13-03202-s001.zip › cancers-1221532-supplementary.pdf]

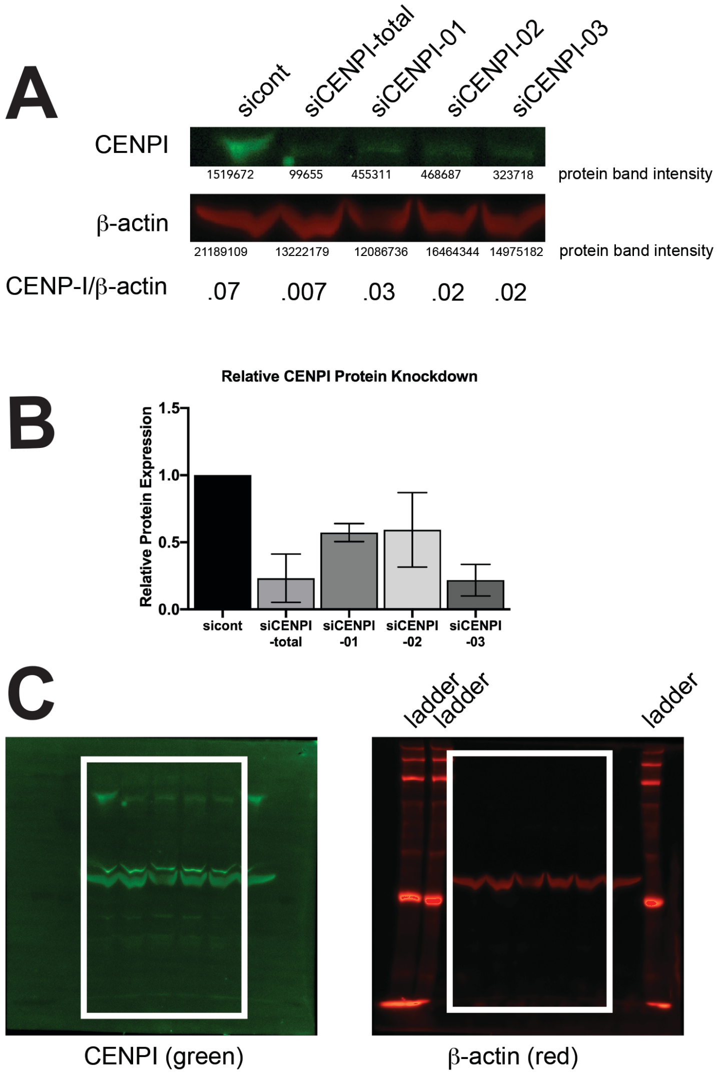

Supplemental Figure S1:Confirmation of CENP-I loss. (A) Steady-state levels of CENP-I was determined by western blot in U251 transfected with control and three unique CENP-I siRNAs. (B) Quantitation of CENP-I western blot analysis was performed using BioRad Image Lab software. (C) Whole gel images of CENP-I and β-actin western blots.

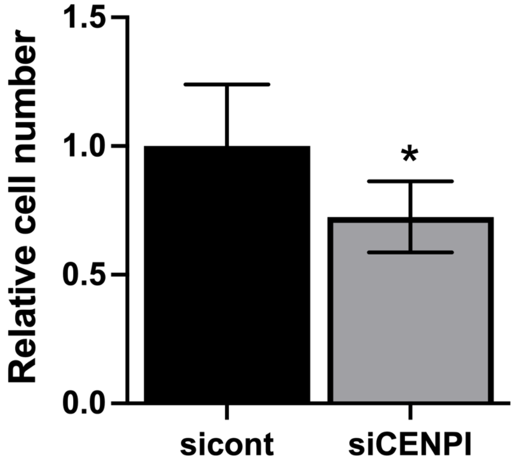

Supplemental Figure S 2: CENP-I loss does not affect cell growth. LN229 cells were transfected with control (cont) and CENP-I siRNAs. Relative cell number was calculated by dividing siRNA treated cells by mock treated cells. Cell growth of 50% or greater reduction is considered detrimental. Statistical analysis was performed using students's t test. *=p<0.05

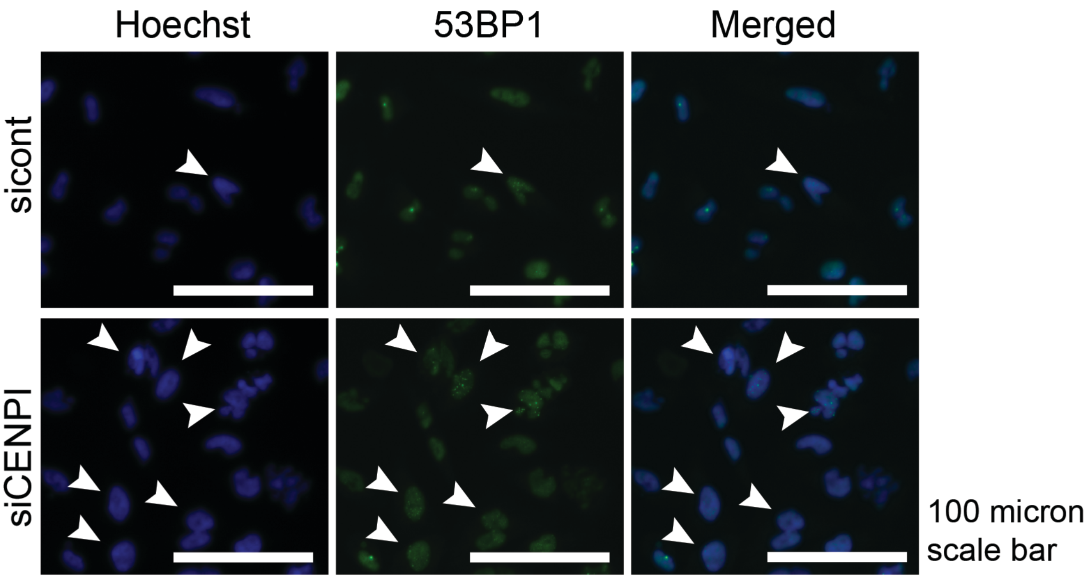

Supplemental Figure S3: Loss of CENP-I induces 53BP1 foci formation. LN229 cells were transfected with control (cont) and CENP-I siRNAs and examined by immunofluorescence for 53BP1 foci formation. Scale bar is equal to 100 microns. Arrows indicate cells with 5+ 53BP1 foci.

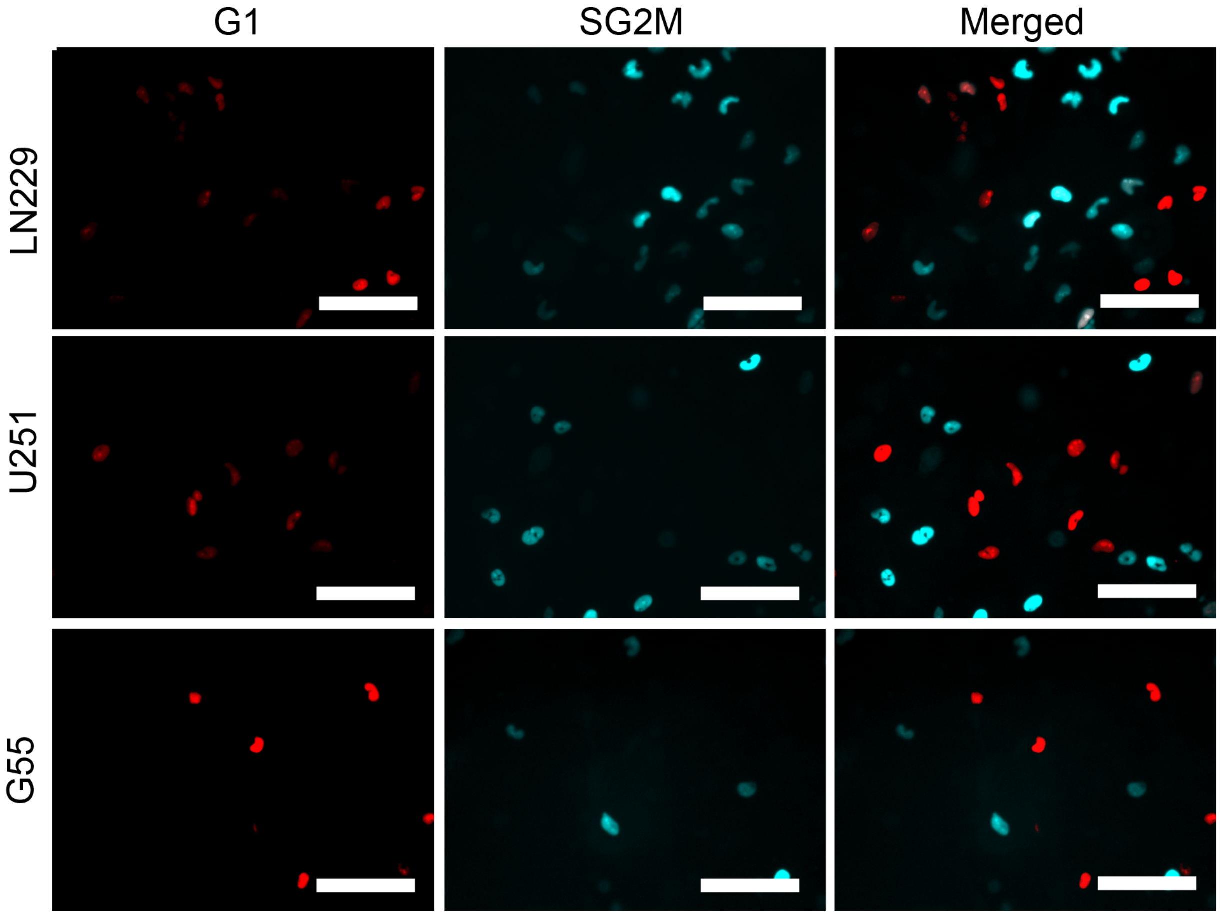

Supplemental Figure S4: Generation of cell-cycle reporter cells. LN229, U251 and G55 established human glioblastoma were transduced with lentiviruses expressing an RFP fused Cdt1 and CYAN fused Geminin. RFP-Cdt1 is expressed in G1 cells and CYAN-Geminin is expressed in S/G2/M cells. Scale bar is 100 micron.

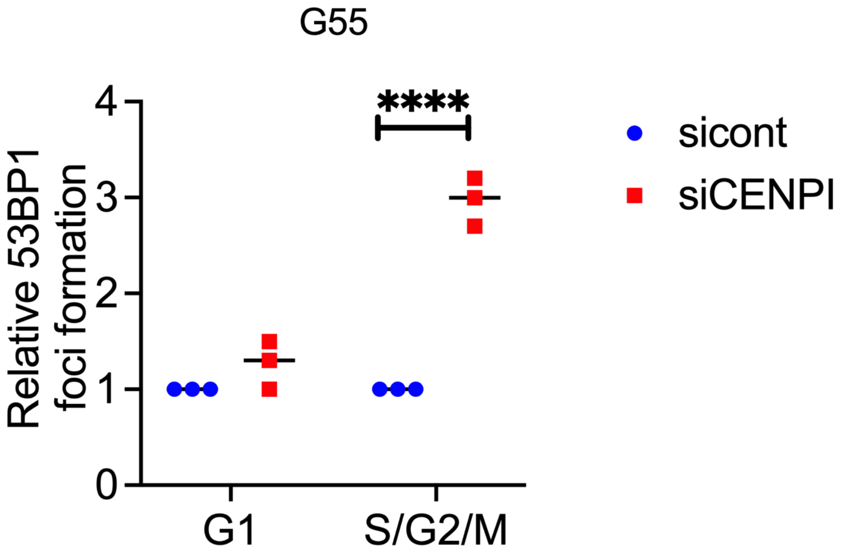

Supplemental Figure S5: Loss of CENP-I results in increased DNA damage in S/G2/M cells. 53BP1 foci was quantitated in G55 G1 and S/G2/M cell with and without CENP-I. Statistical analysis performed using student's t test. ****=$p<0.0001$

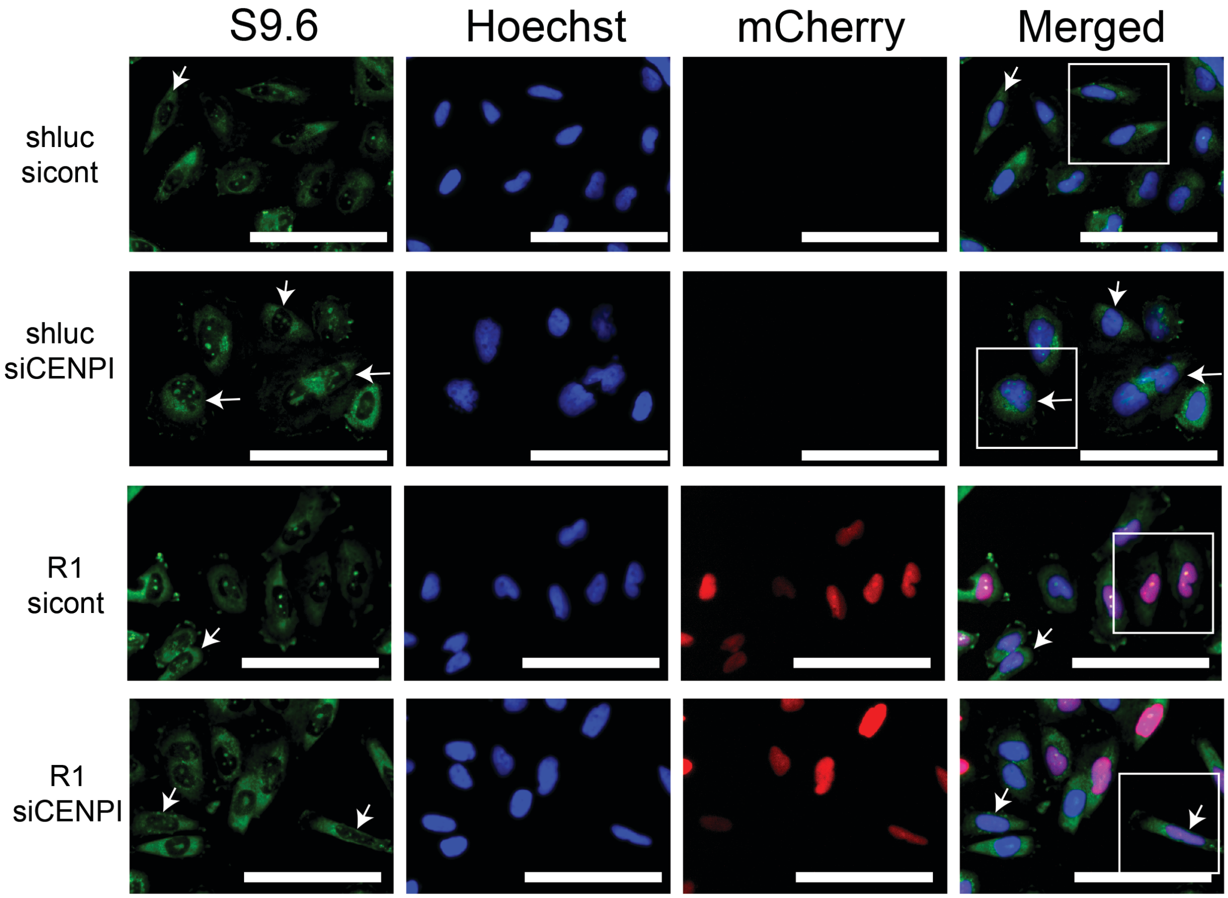

Arrows point to cells with 5+ foci
Scale bar is 100 micron

Supplemental Figure S6: Loss of CENP-I results in RNA:DNA hybrid formation. Nuclear S9.6 foci formation was examined in LN229 control (shluc) and LN229 RNaseH1 expressing (R1) cells exposed to control (NT) and CENPI siRNAs. White box indicates enlarged cells in Figure 6.
